# Posterior reversible encephalopathy syndrome complicating macrophage activation syndrome in a patient with systemic juvenile idiopathic arthritis

**DOI:** 10.1186/1546-0096-12-S1-P228

**Published:** 2014-09-17

**Authors:** Maria Tsinti, Vasiliki Dermentzoglou, Elena Tsitsami

**Affiliations:** 1 Department of Radiology, Children’s Hospital "Aghia Sophia", University of Athens, Athens, Greece; 2 Children's Hospital "Aghia Sofia", University of Athens, Athens, Greece; 3 1st Department of Pediatrics, University of Athens, Athens, Greece

## Introduction

Posterior reversible encephalopathy syndrome (PRES) is a distinct clinical entity presenting with a constellation of neurological symptoms and characteristic neuroimaging findings of posterior cerebral white matter edema. In adults, PRES has been associated with a variety of conditions and predisposing factors, including acute hypertension, preeclampsia or eclampsia, renal disease, sepsis, and exposure to immunosuppressants. The syndrome has been reported less frequently in children, with only one publication reporting the association of PRES with macrophage activation syndrome (MAS) in a patient with systemic onset juvenile idiopathic arthritis (SoJIA).

## Objectives

We report a case of newly diagnosed SoJIA complicated with MAS during the abatement of which developed PRES.

## Methods

Case report.

## Results

A newly diagnosed 8-year-old boy with SoJIA left the hospital on prednizolone (1 mg/kg p.o.), in order to re-examined after 10 days. At that time, he appeared with hemoglobin 104 g/L, leukocytes 17.44x10^9^/L, platelets 406x10^9^/L, ESR 120 mm and serum ferritin 1050 μg/L. One week later and despite his gradual clinical improvement, he re-admitted because he presented persistent high grade fever and rash. On re-admission, laboratory results have shown hemoglobin 96 g/L, leukocytes 5.60x10^9^/L, platelets 208x10^9^/L, ESR 60 mm, serum ferritin 7500 μg/L, fibrinogen 1.23 g/L, LDH 1526 U/L and triglycerides 582 mg/dL. With the tentative diagnosis of MAS the patient administered prednisolone pulses and cyclosporine (75 mg x 2 / day). His clinical picture and laboratory findings were rapidly improved but, on the fifth day from the initiation of treatment, elevation the blood pressure was appeared and tapering of prednisolone and cyclosporine was started. The day after, the patient presented tonic-clonic seizures that necessitated his admission in the intensive care unit for two days. Axial T2-w and FLAIR MRI images demonstrated characteristic of PRES, bilateral and symmetrical high signal intensity lesions involving cortical and subcortical areas of the posterior part of the hemispheres. Prednizolone and cyclosporine discontinued, and the neurological syndrome was completely controlled with antihypertensives and antiepileptics. Two weeks later the patient left the hospital on anakinra, prednisolone (1 mg/kg p.o.) and antiepileptics. After six months, prednisolone has been discontinued and the patient is free of inflammatory symptoms but any attempt to discontinue antiepileptics leads to the relapse of seizures. However, follow up MRI showed complete resolution of previously noted abnormal signal of PRES lesions.

## Conclusion

Approximately 30% of pediatric patients with MAS complicating autoimmune diseases present with neurological manifestations mimicking those of PRES (headaches, seizures, etc.). Therefore, a possible association of MAS with PRES imposes awareness towards prompt radiological examination that will facilitate the early recognition of the last condition and the timely institution of appropriate treatment in order avoid to long-term neurologic deficits.

## Disclosure of interest

None declared.

